# Bis(2,9-dimethyl-1,10-phenanthroline-κ^2^ *N*,*N*′)(10,11,12,13-tetra­hydro­dipyrido[3,2-*a*:2′,3′-*c*]phenazine-κ^2^ *N* ^4^,*N* ^5^)ruthenium(II) bis­(perchlorate) dihydrate

**DOI:** 10.1107/S1600536810002874

**Published:** 2010-01-30

**Authors:** Fu-Hai Wu, Zheng-Zheng Li, Cheng-Hui Zeng, Zhen-Hua Liang, Yun-Jun Liu

**Affiliations:** a School of Public Health, Guangdong Pharmaceutical University, Guangzhou 510006, People’s Republic of China; b School of Pharmacy, Guangdong Pharmaceutical University, Guangzhou 510006, People’s Republic of China

## Abstract

The title compound, [Ru(C_14_H_12_N_2_)_2_(C_18_H_14_N_4_)](ClO_4_)_2_·2H_2_O, consists of an Ru^II^ complex cation, two perchlorate anions and two uncoordinated water mol­ecules. The Ru^II^ ion is chelated by a 10,11,12,13-tetra­hydro­dipyrido[3,2-*a*:2′,3′-*c*]phenazine ligand and two 2,9-dimethyl-1,10-phenanthroline ligands in a distorted octa­hedral geometry. The two uncoord­inated water mol­ecules are disordered over five positions, with an occupancy factor of about 0.4 for each site. A supra­molecular structure is formed by weak π–π inter­actions between neighbouring mol­ecules, with centroid–centroid distances of 3.618 (2) and 3.749 (2) Å.

## Related literature

For general background to ruthenium complexes, see: Abdur-Rashid *et al.* (2002 ▶); Cocchietto & Sava (2000 ▶); Juris *et al.* (1988 ▶); Zorzet *et al.* (2001 ▶). For the synthesis, see: Dickeson & Summers (1970 ▶); Pellegrini & Aldrich-Wright (2003 ▶).
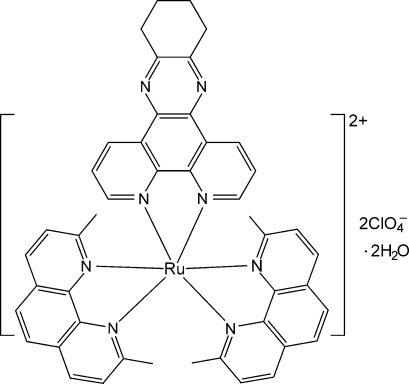


## Experimental

### 

#### Crystal data

[Ru(C_14_H_12_N_2_)_2_(C_18_H_14_N_4_)](ClO_4_)_2_·2H_2_O
*M* *_r_* = 1038.85
Monoclinic, 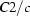

*a* = 25.862 (7) Å
*b* = 24.442 (7) Å
*c* = 18.517 (5) Å
β = 126.229 (5)°
*V* = 9442 (5) Å^3^
*Z* = 8
Mo *K*α radiation
μ = 0.51 mm^−1^
*T* = 291 K
0.28 × 0.22 × 0.20 mm

#### Data collection

Bruker SMART APEX CCD diffractometer
Absorption correction: multi-scan (*SADABS*; Bruker, 2001 ▶) *T*_min_ = 0.87, *T*_max_ = 0.91
21316 measured reflections
9145 independent reflections
7262 reflections with *I* > 2σ(*I*)
*R*_int_ = 0.028

#### Refinement

*R*[*F* ^2^ > 2σ(*F* ^2^)] = 0.050
*wR*(*F* ^2^) = 0.128
*S* = 1.06
9145 reflections
640 parameters
1 restraint
H-atom parameters constrained
Δρ_max_ = 1.33 e Å^−3^
Δρ_min_ = −1.19 e Å^−3^

### 

Data collection: *SMART* (Bruker, 2007 ▶); cell refinement: *SAINT* (Bruker, 2007 ▶); data reduction: *SAINT*; program(s) used to solve structure: *SHELXTL* (Sheldrick, 2008 ▶); program(s) used to refine structure: *SHELXTL*; molecular graphics: *SHELXTL*; software used to prepare material for publication: *SHELXTL*.

## Supplementary Material

Crystal structure: contains datablocks global, I. DOI: 10.1107/S1600536810002874/hy2273sup1.cif

Structure factors: contains datablocks I. DOI: 10.1107/S1600536810002874/hy2273Isup2.hkl

Additional supplementary materials:  crystallographic information; 3D view; checkCIF report

## Figures and Tables

**Table 1:** Selected bond lengths (Å)

Ru1—N1	2.068 (3)
Ru1—N2	2.061 (3)
Ru1—N5	2.094 (3)
Ru1—N6	2.108 (3)
Ru1—N7	2.106 (3)
Ru1—N8	2.108 (3)

**Fig. 1.**
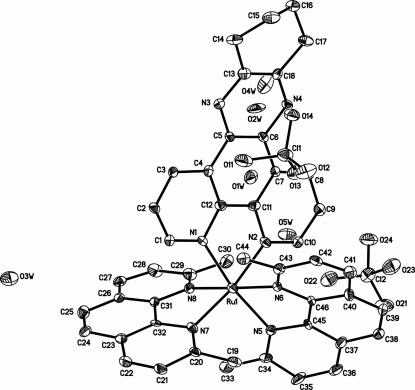
Molecular structure of the title compound. Displacement ellipsoids are drawn at the 30% probability level. H atoms have been omitted for clarity. The two disordered water molecules occupy five different positions, O1W, O2W, O3W, O4W and O5W.

**Fig. 2.**
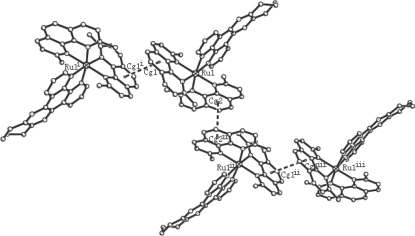
A view of π–π interactions between the neighbouring molecules. H atoms have been omitted for clarity. [Symmetry codes: (i) 1/2-x, 3/2-y, z; (ii) -x, 2-y, -z; (iii) -1/2+x, 1/2+y, z.]

**Fig. 3.**
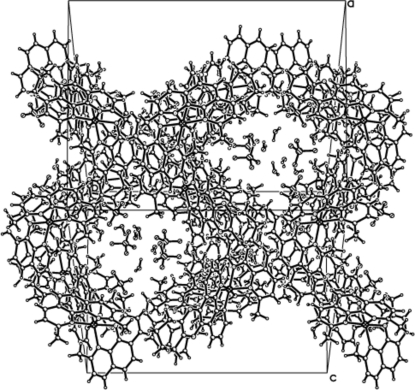
A view of the supramolecular channel structure down [101] direction.

## supplementary crystallographic information

### Comment

Ruthenium(II) complexes have been investigated extensively during the past two decades due to their rich photochemical and photophysical properties (Juris *et al.*, 1988). Ruthenium complexes have been applied in many important fields, such as catalysis, antitumor drugs and DNA structural probe. [Ru(*R*-binap)(H)_2_(tmen)] (binap = bidentate phosphine; tmen = H_2_NCMe_2_CMe_2_NH_2_) can catalyze the hydrogenation of ketone (Abdur-Rashid *et al.*, 2002). Complex ImH[*trans*-Ru(III)Cl4(DMSO)Im] (Im = imidazole; DMSO = dimethyl sulfoxide) was the first ruthenium complex to enter clinical trials against metastases (Cocchietto & Sava, 2000; Zorzet *et al.*, 2001). In an attempt to obtain more insight into the structural properties of such ruthenium(II) complexes, we present here the crystal structure of the title complex.

The crystal structure of the title compound reveals that all the bond lengths and angles have normal values (Table 1). There are one Ru^II^ complex cation, two perchlorate anions and two uncoordinated water molecules per asymmetric unit (Fig. 1). The two uncoordinated water molecules are disordered over five different positions and theirs site occupancy factors are 0.392 (8), 0.410 (9), 0.388 (9), 0.402 (9), and 0.408 (9) for O1W, O2W, O3W, O4W, and O5W, respectively. The Ru^II^ ion is in a distorted octahedral environment, coordinated by N5, N6, N7, N8 from two 2,9-dimethyl-1,10-phenanthroline (dmp) ligands, and N1, N2 from one 10,11,12,13-tetrahydrodipyrido[3,2-a:2',3'-c]phenazine (dpqc) ligand (Fig. 1). The weak π–π interactions between the neighbouring molecules are occurred (Fig. 2). The perpendicular distance of *Cg*1···*Cg*1^i^ from face to face is 3.50 (2) Å and the centroid–centroid distance of *Cg*1···*Cg*1^i^ is 3.749 (2) Å [*Cg*1 is the centroid of C23, C24, C25, C26, C31 and C32 ring; symmetry code: (i) 1/2-x, 3/2-y, z]. The other perpendicular distance of *Cg*2···*Cg*2^ii^ from face to face is 3.48 (2) Å and the centroid–centroid distance of *Cg*2···*Cg*2^ii^ is 3.618 (2) Å [*Cg*2 is the centroid of C37, C38, C39, C40, C45 and C46 ring; symmetry code: (ii) -x, 2-y, -z]. Through π–π interactions, a supramolecular structure is formed. There are channels along the [1 0 1] direction filled with the uncoordinated water molecules and perchlorate anions (Fig. 3).

### Experimental

Ligand dpqc was prepared by a modified method reported in the literature (Dickeson & Summers, 1970). A mixture of phenanthroline-5,6-diamine (0.210 g, 1 mmol), 1,2-cyclohexanedione (0.112 g, 1 mmol) and glacial acetic acid (30 cm^3^) was refluxed with stirring for 6 h. The cooled solution was diluted with water and neutralized with concentrated aqueous ammonia. A pale yellow-green precipitate was obtained. The product was recrystallized from methanol to give pale yellow-green powders.

The title complex was synthesized by the modified method (Pellegrini & Aldrich-Wright, 2003). A mixture of *cis*-[Ru(dmp)_2_Cl_2_].2H_2_O (0.288 g, 0.5 mmol) and dpqc (0.161 g, 0.5 mmol) in EtOH (40 cm^3^) was refluxed under argon for 8 h to give a clear red solution. Upon cooling, a red precipitate was obtained by dropwise addition of saturated aqueous NaClO_4_ solution. The crude product was purified by column chromatography on a neutral alumina with CH_3_CN-toluene (v/v 3:1) as eluent. The mainly brown red band was collected. The solvent was removed under reduced pressure and a red powder was obtained. Red single crystals of the title complex suitable for X-ray crystallographic study were obtained from acetonitrile and ethanol (v/v 1:3) at room temperature.

### Refinement

In the asymmetric unit there are two disordered water molecules. They occupy five different positions and theirs site occupancy factors were refined with free variable and validated as 0.392 (8), 0.410 (9), 0.388 (9), 0.402 (9) and 0.408 (9) for O1W, O2W, O3W, O4W, and O5W, respectively.

H atoms on C atoms were positioned geometrically and refined as riding atoms, with C—H = 0.93 (CH), 0.97 (CH_2_) and 0.96 (CH_3_) Å and with *U*_iso_(H) = 1.2(1.5 for methyl)*U*_eq_(C). H atoms of water molecules were determined based on difference Fourier maps and possible hydrogen bonding scheme and refined as riding, with *U*_iso_(H) = 1.2*U*_eq_(O). The highest residual electron density was found 1.14 Å from O3W and the deepest hole 0.56 Å from Ru1.

### Crystal data

**Table:**

[Ru(C_14_H_12_N_2_)_2_(C_18_H_14_N_4_)](ClO_4_)_2_·2H_2_O	*F*(000) = 4256
*M**_r_* = 1038.85	*D*_x_ = 1.462 Mg m^−^^3^
Monoclinic, *C*2/*c*	Mo *K*α radiation, λ = 0.71073 Å
Hall symbol: -C 2yc	Cell parameters from 950 reflections
*a* = 25.862 (7) Å	θ = 2.3–26.5°
*b* = 24.442 (7) Å	µ = 0.51 mm^−^^1^
*c* = 18.517 (5) Å	*T* = 291 K
β = 126.229 (5)°	Block, red
*V* = 9442 (5) Å^3^	0.28 × 0.22 × 0.20 mm
*Z* = 8	

### Data collection

**Table:**

Bruker SMART APEX CCD diffractometer	9145 independent reflections
Radiation source: sealed tube	7262 reflections with *I* > 2σ(*I*)
graphite	*R*_int_ = 0.028
φ and ω scans	θ_max_ = 26.0°, θ_min_ = 1.3°
Absorption correction: multi-scan (*SADABS*; Bruker, 2001)	*h* = −31→24
*T*_min_ = 0.87, *T*_max_ = 0.91	*k* = −29→30
21316 measured reflections	*l* = −21→22

### Refinement

**Table:**

Refinement on *F*^2^	Primary atom site location: structure-invariant direct methods
Least-squares matrix: full	Secondary atom site location: difference Fourier map
*R*[*F*^2^ > 2σ(*F*^2^)] = 0.050	Hydrogen site location: inferred from neighbouring sites
*wR*(*F*^2^) = 0.128	H-atom parameters constrained
*S* = 1.06	*w* = 1/[σ^2^(*F*_o_^2^) + (0.08*P*)^2^ + 1.99*P*] where *P* = (*F*_o_^2^ + 2*F*_c_^2^)/3
9145 reflections	(Δ/σ)_max_ < 0.001
640 parameters	Δρ_max_ = 1.33 e Å^−^^3^
1 restraint	Δρ_min_ = −1.19 e Å^−^^3^

### Fractional atomic coordinates and isotropic or equivalent isotropic displacement parameters (Å^2^)

**Table:**

	*x*	*y*	*z*	*U*_iso_*/*U*_eq_	Occ. (<1)
C1	0.34039 (15)	0.95304 (14)	0.1570 (2)	0.0265 (7)	
H1	0.3230	0.9362	0.1021	0.032*	
C2	0.39680 (15)	0.98077 (12)	0.1952 (2)	0.0248 (6)	
H2	0.4162	0.9828	0.1660	0.030*	
C3	0.42516 (14)	1.00603 (12)	0.2782 (2)	0.0230 (6)	
H3	0.4634	1.0253	0.3053	0.028*	
C4	0.39279 (14)	1.00109 (14)	0.3208 (2)	0.0257 (7)	
C5	0.41649 (14)	1.02549 (12)	0.4027 (2)	0.0221 (6)	
C6	0.38381 (14)	1.01967 (12)	0.44067 (19)	0.0197 (6)	
C7	0.32742 (13)	0.98944 (12)	0.39676 (19)	0.0202 (6)	
C8	0.29292 (15)	0.97934 (14)	0.4343 (2)	0.0293 (7)	
H8	0.3087	0.9922	0.4911	0.035*	
C9	0.23564 (16)	0.95010 (13)	0.3846 (2)	0.0284 (7)	
H9	0.2122	0.9440	0.4072	0.034*	
C10	0.21420 (15)	0.93023 (14)	0.3015 (2)	0.0269 (7)	
H10	0.1756	0.9111	0.2692	0.032*	
C11	0.30372 (14)	0.96503 (13)	0.3149 (2)	0.0256 (6)	
C12	0.33640 (15)	0.97086 (13)	0.2769 (2)	0.0253 (6)	
C13	0.48347 (16)	1.09070 (13)	0.5105 (2)	0.0295 (7)	
C14	0.53606 (17)	1.13189 (15)	0.5449 (2)	0.0361 (8)	
H14A	0.5353	1.1462	0.4955	0.043*	
H14B	0.5772	1.1143	0.5866	0.043*	
C15	0.52801 (19)	1.17815 (15)	0.5913 (3)	0.0391 (9)	
H15A	0.5643	1.2028	0.6176	0.047*	
H15B	0.4895	1.1986	0.5480	0.047*	
C16	0.52340 (17)	1.15688 (15)	0.6621 (2)	0.0338 (7)	
H16A	0.5193	1.1875	0.6917	0.041*	
H16B	0.5629	1.1379	0.7064	0.041*	
C17	0.46627 (17)	1.11736 (14)	0.6269 (2)	0.0306 (7)	
H17A	0.4763	1.0930	0.6749	0.037*	
H17B	0.4287	1.1385	0.6093	0.037*	
C18	0.45097 (14)	1.08379 (12)	0.5484 (2)	0.0232 (6)	
C19	0.11611 (16)	0.96086 (14)	−0.0851 (2)	0.0325 (7)	
H19A	0.1068	0.9555	−0.0425	0.049*	
H19B	0.0785	0.9531	−0.1442	0.049*	
H19C	0.1290	0.9981	−0.0823	0.049*	
C20	0.16888 (15)	0.92348 (13)	−0.0636 (2)	0.0247 (6)	
C21	0.18028 (16)	0.91234 (14)	−0.1295 (2)	0.0295 (7)	
H21	0.1551	0.9297	−0.1848	0.035*	
C22	0.22522 (16)	0.87833 (15)	−0.1136 (2)	0.0328 (7)	
H22	0.2337	0.8742	−0.1555	0.039*	
C23	0.26122 (14)	0.84758 (13)	−0.0320 (2)	0.0260 (7)	
C24	0.30726 (14)	0.80762 (15)	−0.0119 (2)	0.0306 (7)	
H24	0.3186	0.8025	−0.0506	0.037*	
C25	0.33534 (17)	0.77638 (14)	0.0632 (2)	0.0315 (7)	
H25	0.3645	0.7492	0.0745	0.038*	
C26	0.31950 (14)	0.78568 (12)	0.1254 (2)	0.0235 (6)	
C27	0.34215 (15)	0.75126 (13)	0.1991 (2)	0.0281 (7)	
H27	0.3701	0.7227	0.2117	0.034*	
C28	0.32210 (18)	0.76055 (14)	0.2525 (3)	0.0372 (8)	
H28	0.3353	0.7373	0.3002	0.045*	
C29	0.28163 (16)	0.80525 (13)	0.2347 (2)	0.0294 (7)	
C30	0.26241 (17)	0.81254 (14)	0.2962 (3)	0.0337 (8)	
H30B	0.2959	0.8312	0.3494	0.040*	
H30C	0.2552	0.7774	0.3119	0.040*	
H30A	0.2237	0.8337	0.2667	0.040*	
C31	0.27856 (14)	0.82747 (12)	0.1108 (2)	0.0236 (6)	
C32	0.24751 (13)	0.85874 (13)	0.0299 (2)	0.0233 (6)	
C33	0.12338 (19)	0.79828 (15)	0.0501 (3)	0.0431 (9)	
H33A	0.1544	0.8112	0.0415	0.065*	
H33B	0.0900	0.7790	−0.0025	0.065*	
H33C	0.1438	0.7741	0.1007	0.065*	
C34	0.09535 (16)	0.84562 (14)	0.0664 (2)	0.0332 (8)	
C35	0.03673 (16)	0.83525 (16)	0.0575 (3)	0.0441 (10)	
H35	0.0172	0.8011	0.0395	0.053*	
C36	0.01039 (16)	0.87696 (16)	0.0764 (3)	0.0398 (9)	
H36	−0.0253	0.8706	0.0757	0.048*	
C37	0.03902 (15)	0.93059 (14)	0.0970 (2)	0.0307 (7)	
C38	0.01269 (15)	0.97619 (15)	0.1127 (2)	0.0307 (7)	
H38	−0.0230	0.9711	0.1126	0.037*	
C39	0.03840 (15)	1.02695 (14)	0.1278 (2)	0.0305 (7)	
H39	0.0218	1.0561	0.1406	0.037*	
C40	0.09095 (15)	1.03482 (13)	0.1239 (2)	0.0275 (7)	
C41	0.11770 (17)	1.08719 (14)	0.1297 (3)	0.0354 (8)	
H41	0.1022	1.1182	0.1403	0.042*	
C42	0.16591 (16)	1.09204 (14)	0.1199 (2)	0.0288 (7)	
H42	0.1814	1.1265	0.1207	0.035*	
C43	0.19258 (14)	1.04466 (13)	0.1085 (2)	0.0241 (6)	
C44	0.24055 (16)	1.05216 (14)	0.0908 (2)	0.0303 (7)	
H44A	0.2804	1.0635	0.1448	0.046*	
H44B	0.2259	1.0796	0.0456	0.046*	
H44C	0.2466	1.0182	0.0706	0.046*	
C45	0.09540 (15)	0.93645 (13)	0.1032 (2)	0.0237 (6)	
C46	0.12114 (14)	0.98929 (13)	0.1149 (2)	0.0223 (6)	
Cl1	0.33340 (4)	0.15639 (4)	0.28808 (6)	0.0418 (2)	
Cl2	0.39352 (4)	0.33611 (4)	0.11989 (6)	0.0400 (2)	
N1	0.30797 (12)	0.94835 (11)	0.19321 (17)	0.0231 (5)	
N2	0.24546 (12)	0.93662 (11)	0.26341 (17)	0.0228 (5)	
N3	0.46872 (12)	1.06006 (12)	0.44001 (18)	0.0275 (6)	
N4	0.40222 (12)	1.04887 (10)	0.51642 (16)	0.0220 (5)	
N5	0.12519 (12)	0.89381 (11)	0.09329 (17)	0.0217 (5)	
N6	0.17299 (11)	0.99366 (11)	0.11172 (16)	0.0218 (5)	
N7	0.20461 (13)	0.89915 (10)	0.01584 (18)	0.0226 (5)	
N8	0.26208 (12)	0.83945 (10)	0.16727 (18)	0.0225 (5)	
O1W	0.3498 (3)	0.8384 (3)	0.5030 (5)	0.040 (2)	0.392 (8)
H1WD	0.3768	0.8630	0.5364	0.048*	0.392 (8)
H1WC	0.3370	0.8270	0.5333	0.048*	0.392 (8)
O2W	0.4328 (3)	0.9140 (3)	0.6056 (5)	0.050 (2)	0.410 (9)
H2WA	0.4204	0.9452	0.5662	0.060*	0.410 (9)
H2WB	0.4603	0.9198	0.6698	0.060*	0.410 (9)
O3W	0.4565 (4)	0.6672 (3)	0.1768 (5)	0.050 (3)	0.388 (9)
H3WA	0.4897	0.6752	0.1799	0.060*	0.388 (9)
H3WB	0.4584	0.6339	0.1915	0.060*	0.388 (9)
O4W	0.4616 (3)	0.7646 (2)	0.8166 (5)	0.049 (3)	0.402 (9)
H4WD	0.4612	0.7577	0.7713	0.059*	0.402 (9)
H4WC	0.4262	0.7791	0.7995	0.059*	0.402 (9)
O5W	0.2432 (4)	0.7111 (3)	0.5185 (6)	0.058 (3)	0.408 (9)
H5WD	0.2788	0.7199	0.5671	0.070*	0.408 (9)
H5WA	0.2140	0.7113	0.5264	0.070*	0.408 (9)
O11	0.36730 (14)	0.12927 (12)	0.2590 (2)	0.0524 (7)	
O12	0.28994 (14)	0.19350 (12)	0.2330 (2)	0.0541 (8)	
O13	0.30154 (13)	0.11268 (12)	0.2990 (2)	0.0502 (7)	
O14	0.37913 (13)	0.17688 (12)	0.37388 (19)	0.0484 (7)	
O21	0.44873 (15)	0.30330 (13)	0.1473 (2)	0.0587 (8)	
O22	0.36080 (13)	0.32210 (13)	0.1550 (2)	0.0485 (7)	
O23	0.40737 (14)	0.39303 (13)	0.1303 (2)	0.0569 (8)	
O24	0.34700 (14)	0.33037 (12)	0.0261 (2)	0.0531 (8)	
Ru1	0.217470 (12)	0.916700 (10)	0.136870 (17)	0.02439 (10)	

### Atomic displacement parameters (Å^2^)

**Table:**

	*U*^11^	*U*^22^	*U*^33^	*U*^12^	*U*^13^	*U*^23^
C1	0.0229 (15)	0.0325 (17)	0.0266 (16)	−0.0001 (12)	0.0161 (13)	−0.0040 (13)
C2	0.0279 (15)	0.0173 (15)	0.0252 (16)	0.0026 (11)	0.0135 (13)	−0.0041 (12)
C3	0.0208 (14)	0.0180 (14)	0.0285 (17)	0.0008 (11)	0.0136 (13)	−0.0046 (12)
C4	0.0215 (15)	0.0319 (17)	0.0255 (16)	0.0035 (13)	0.0149 (13)	−0.0017 (13)
C5	0.0212 (14)	0.0196 (14)	0.0215 (15)	0.0038 (11)	0.0104 (12)	−0.0041 (12)
C6	0.0249 (14)	0.0184 (14)	0.0147 (14)	0.0043 (11)	0.0110 (12)	0.0029 (11)
C7	0.0222 (14)	0.0216 (14)	0.0147 (14)	0.0086 (11)	0.0097 (12)	0.0050 (11)
C8	0.0275 (16)	0.0361 (18)	0.0254 (17)	0.0011 (13)	0.0164 (14)	−0.0044 (14)
C9	0.0309 (16)	0.0257 (16)	0.0250 (17)	0.0096 (13)	0.0144 (14)	0.0049 (13)
C10	0.0216 (15)	0.0328 (17)	0.0242 (16)	0.0031 (13)	0.0124 (13)	−0.0013 (13)
C11	0.0223 (14)	0.0287 (17)	0.0172 (14)	0.0031 (12)	0.0070 (12)	0.0041 (13)
C12	0.0251 (15)	0.0283 (16)	0.0175 (15)	0.0036 (12)	0.0097 (12)	0.0017 (13)
C13	0.0294 (17)	0.0229 (16)	0.0286 (18)	0.0013 (13)	0.0129 (14)	0.0011 (13)
C14	0.0310 (17)	0.043 (2)	0.0239 (17)	−0.0144 (15)	0.0104 (14)	−0.0022 (15)
C15	0.039 (2)	0.0291 (18)	0.036 (2)	−0.0143 (15)	0.0153 (17)	0.0021 (15)
C16	0.0322 (17)	0.0341 (19)	0.0287 (18)	−0.0065 (14)	0.0146 (15)	−0.0054 (14)
C17	0.0365 (18)	0.0282 (17)	0.0178 (15)	−0.0041 (14)	0.0109 (14)	0.0022 (13)
C18	0.0190 (14)	0.0200 (15)	0.0233 (16)	0.0109 (11)	0.0085 (12)	0.0049 (12)
C19	0.0340 (18)	0.0260 (17)	0.0244 (17)	−0.0014 (14)	0.0100 (14)	−0.0053 (13)
C20	0.0262 (15)	0.0252 (15)	0.0249 (16)	−0.0102 (12)	0.0163 (13)	−0.0104 (13)
C21	0.0328 (17)	0.0373 (19)	0.0172 (15)	−0.0031 (14)	0.0140 (14)	−0.0022 (13)
C22	0.0312 (17)	0.0369 (19)	0.0237 (17)	0.0011 (14)	0.0127 (14)	−0.0047 (14)
C23	0.0232 (15)	0.0221 (15)	0.0320 (17)	−0.0093 (12)	0.0159 (13)	−0.0092 (13)
C24	0.0183 (15)	0.045 (2)	0.0253 (17)	−0.0008 (13)	0.0112 (14)	−0.0006 (14)
C25	0.0314 (17)	0.0264 (17)	0.038 (2)	0.0024 (13)	0.0213 (16)	0.0012 (14)
C26	0.0181 (14)	0.0148 (14)	0.0325 (17)	−0.0044 (11)	0.0120 (13)	−0.0094 (12)
C27	0.0212 (15)	0.0232 (16)	0.0306 (17)	−0.0041 (12)	0.0101 (13)	−0.0027 (13)
C28	0.045 (2)	0.0245 (17)	0.0316 (19)	0.0066 (15)	0.0172 (17)	0.0041 (15)
C29	0.0352 (17)	0.0167 (15)	0.0314 (18)	−0.0011 (13)	0.0170 (15)	−0.0008 (13)
C30	0.0297 (17)	0.0250 (17)	0.046 (2)	−0.0026 (13)	0.0222 (17)	0.0018 (15)
C31	0.0181 (14)	0.0208 (15)	0.0322 (17)	−0.0060 (11)	0.0150 (13)	−0.0115 (12)
C32	0.0174 (14)	0.0250 (15)	0.0183 (14)	−0.0068 (11)	0.0055 (12)	−0.0137 (12)
C33	0.0352 (19)	0.0294 (19)	0.056 (3)	−0.0111 (15)	0.0223 (19)	−0.0197 (18)
C34	0.0239 (15)	0.0317 (18)	0.0357 (19)	−0.0109 (14)	0.0130 (14)	−0.0163 (15)
C35	0.0169 (16)	0.036 (2)	0.062 (3)	−0.0065 (14)	0.0137 (17)	−0.0127 (18)
C36	0.0186 (15)	0.042 (2)	0.045 (2)	−0.0050 (14)	0.0114 (15)	−0.0104 (17)
C37	0.0190 (15)	0.0312 (18)	0.039 (2)	−0.0007 (13)	0.0154 (14)	−0.0040 (15)
C38	0.0218 (15)	0.040 (2)	0.0242 (16)	0.0059 (14)	0.0104 (13)	−0.0008 (14)
C39	0.0225 (15)	0.0364 (19)	0.0323 (18)	0.0060 (13)	0.0159 (14)	−0.0051 (15)
C40	0.0284 (16)	0.0290 (17)	0.0277 (17)	0.0071 (13)	0.0180 (14)	−0.0001 (14)
C41	0.0334 (18)	0.0218 (17)	0.040 (2)	0.0056 (13)	0.0155 (16)	−0.0050 (14)
C42	0.0330 (17)	0.0245 (16)	0.0213 (16)	−0.0009 (13)	0.0118 (13)	−0.0058 (13)
C43	0.0231 (15)	0.0218 (15)	0.0201 (15)	0.0065 (12)	0.0087 (12)	0.0061 (12)
C44	0.0302 (17)	0.0254 (17)	0.0297 (18)	0.0000 (13)	0.0146 (14)	0.0011 (14)
C45	0.0240 (15)	0.0317 (16)	0.0207 (15)	−0.0005 (13)	0.0161 (12)	−0.0093 (13)
C46	0.0190 (13)	0.0293 (16)	0.0196 (15)	0.0018 (12)	0.0119 (12)	−0.0083 (12)
Cl1	0.0395 (5)	0.0351 (5)	0.0338 (5)	0.0050 (4)	0.0123 (4)	0.0008 (4)
Cl2	0.0356 (4)	0.0457 (5)	0.0371 (5)	0.0017 (4)	0.0206 (4)	0.0089 (4)
N1	0.0227 (12)	0.0264 (14)	0.0196 (13)	−0.0017 (10)	0.0122 (10)	−0.0075 (10)
N2	0.0289 (13)	0.0204 (12)	0.0229 (13)	−0.0052 (10)	0.0174 (11)	−0.0041 (10)
N3	0.0209 (13)	0.0342 (15)	0.0229 (14)	0.0024 (11)	0.0104 (11)	−0.0037 (12)
N4	0.0236 (12)	0.0256 (13)	0.0147 (12)	0.0011 (10)	0.0101 (10)	−0.0003 (10)
N5	0.0204 (12)	0.0207 (13)	0.0213 (13)	0.0022 (10)	0.0108 (10)	0.0010 (10)
N6	0.0185 (12)	0.0260 (13)	0.0195 (13)	0.0013 (10)	0.0105 (10)	−0.0049 (10)
N7	0.0301 (13)	0.0163 (12)	0.0257 (14)	−0.0058 (10)	0.0188 (11)	−0.0050 (10)
N8	0.0207 (12)	0.0193 (13)	0.0263 (14)	0.0001 (10)	0.0132 (11)	−0.0030 (10)
O1W	0.033 (4)	0.028 (4)	0.051 (5)	0.005 (3)	0.020 (3)	−0.002 (3)
O2W	0.027 (3)	0.040 (4)	0.048 (4)	−0.014 (3)	0.003 (3)	−0.014 (3)
O3W	0.046 (4)	0.044 (5)	0.048 (5)	−0.008 (3)	0.021 (4)	−0.001 (3)
O4W	0.045 (4)	0.023 (3)	0.071 (5)	0.023 (3)	0.030 (4)	0.029 (3)
O5W	0.047 (4)	0.047 (5)	0.069 (6)	−0.031 (3)	0.027 (4)	−0.035 (4)
O11	0.0510 (17)	0.0456 (17)	0.0435 (17)	−0.0109 (13)	0.0185 (14)	−0.0143 (13)
O12	0.0442 (16)	0.0383 (15)	0.0522 (18)	−0.0041 (12)	0.0133 (14)	0.0193 (13)
O13	0.0409 (15)	0.0458 (17)	0.0411 (16)	0.0064 (13)	0.0117 (13)	0.0170 (13)
O14	0.0421 (15)	0.0451 (16)	0.0411 (16)	−0.0032 (12)	0.0153 (13)	−0.0170 (13)
O21	0.0484 (17)	0.0530 (19)	0.058 (2)	0.0214 (14)	0.0222 (16)	0.0207 (16)
O22	0.0397 (15)	0.0542 (18)	0.0471 (17)	0.0048 (13)	0.0232 (14)	−0.0085 (14)
O23	0.0426 (16)	0.0523 (19)	0.055 (2)	−0.0084 (14)	0.0179 (15)	−0.0131 (15)
O24	0.0443 (16)	0.0499 (17)	0.0413 (17)	0.0084 (13)	0.0121 (14)	0.0133 (14)
Ru1	0.02504 (15)	0.02389 (15)	0.02189 (15)	−0.00006 (10)	0.01257 (12)	−0.00463 (10)

### Geometric parameters (Å, °)

**Table:**

C1—N1	1.353 (4)	C29—N8	1.329 (4)
C1—C2	1.367 (4)	C29—C30	1.498 (5)
C1—H1	0.9300	C30—H30B	0.9600
C2—C3	1.396 (4)	C30—H30C	0.9600
C2—H2	0.9300	C30—H30A	0.9600
C3—C4	1.456 (4)	C31—N8	1.373 (4)
C3—H3	0.9300	C31—C32	1.432 (5)
C4—C5	1.390 (4)	C32—N7	1.390 (4)
C4—C12	1.390 (5)	C33—C34	1.488 (5)
C5—N3	1.383 (4)	C33—H33A	0.9600
C5—C6	1.390 (4)	C33—H33B	0.9600
C6—N4	1.384 (4)	C33—H33C	0.9600
C6—C7	1.390 (4)	C34—N5	1.333 (4)
C7—C11	1.390 (4)	C34—C35	1.448 (5)
C7—C8	1.439 (4)	C35—C36	1.380 (5)
C8—C9	1.393 (5)	C35—H35	0.9300
C8—H8	0.9300	C36—C37	1.442 (5)
C9—C10	1.381 (5)	C36—H36	0.9300
C9—H9	0.9300	C37—C45	1.401 (4)
C10—N2	1.359 (4)	C37—C38	1.423 (5)
C10—H10	0.9300	C38—C39	1.356 (5)
C11—C12	1.390 (4)	C38—H38	0.9300
C11—N2	1.401 (4)	C39—C40	1.417 (5)
C12—N1	1.378 (4)	C39—H39	0.9300
C13—N3	1.349 (4)	C40—C46	1.424 (4)
C13—C18	1.389 (5)	C40—C41	1.428 (5)
C13—C14	1.498 (5)	C41—C42	1.367 (5)
C14—C15	1.507 (6)	C41—H41	0.9300
C14—H14A	0.9700	C42—C43	1.426 (4)
C14—H14B	0.9700	C42—H42	0.9300
C15—C16	1.478 (5)	C43—N6	1.360 (4)
C15—H15A	0.9700	C43—C44	1.469 (5)
C15—H15B	0.9700	C44—H44A	0.9600
C16—C17	1.550 (5)	C44—H44B	0.9600
C16—H16A	0.9700	C44—H44C	0.9600
C16—H16B	0.9700	C45—N5	1.372 (4)
C17—C18	1.504 (5)	C45—C46	1.410 (5)
C17—H17A	0.9700	C46—N6	1.381 (4)
C17—H17B	0.9700	Cl1—O12	1.331 (3)
C18—N4	1.336 (4)	Cl1—O14	1.397 (3)
C19—C20	1.488 (5)	Cl1—O11	1.434 (3)
C19—H19A	0.9600	Cl1—O13	1.435 (3)
C19—H19B	0.9600	Cl2—O22	1.380 (3)
C19—H19C	0.9600	Cl2—O24	1.419 (3)
C20—N7	1.329 (4)	Cl2—O23	1.421 (3)
C20—C21	1.439 (4)	Cl2—O21	1.443 (3)
C21—C22	1.313 (5)	Ru1—N1	2.068 (3)
C21—H21	0.9300	Ru1—N2	2.061 (3)
C22—C23	1.432 (5)	Ru1—N5	2.094 (3)
C22—H22	0.9300	Ru1—N6	2.108 (3)
C23—C24	1.409 (5)	Ru1—N7	2.106 (3)
C23—C32	1.412 (4)	Ru1—N8	2.108 (3)
C24—C25	1.359 (5)	O1W—H1WD	0.8499
C24—H24	0.9300	O1W—H1WC	0.8498
C25—C26	1.450 (5)	O2W—H2WA	0.9700
C25—H25	0.9300	O2W—H2WB	0.9700
C26—C31	1.379 (4)	O3W—H3WA	0.8498
C26—C27	1.403 (5)	O3W—H3WB	0.8499
C27—C28	1.379 (5)	O4W—H4WD	0.8499
C27—H27	0.9300	O4W—H4WC	0.8500
C28—C29	1.411 (5)	O5W—H5WD	0.8500
C28—H28	0.9300	O5W—H5WA	0.8500
			
N1—C1—C2	124.8 (3)	H30C—C30—H30A	109.5
N1—C1—H1	117.6	N8—C31—C26	123.9 (3)
C2—C1—H1	117.6	N8—C31—C32	116.6 (3)
C1—C2—C3	119.6 (3)	C26—C31—C32	119.4 (3)
C1—C2—H2	120.2	N7—C32—C23	122.7 (3)
C3—C2—H2	120.2	N7—C32—C31	117.9 (3)
C2—C3—C4	117.6 (3)	C23—C32—C31	119.4 (3)
C2—C3—H3	121.2	C34—C33—H33A	109.5
C4—C3—H3	121.2	C34—C33—H33B	109.5
C5—C4—C12	120.0 (3)	H33A—C33—H33B	109.5
C5—C4—C3	122.1 (3)	C34—C33—H33C	109.5
C12—C4—C3	117.9 (3)	H33A—C33—H33C	109.5
N3—C5—C4	118.6 (3)	H33B—C33—H33C	109.5
N3—C5—C6	121.1 (3)	N5—C34—C35	122.2 (3)
C4—C5—C6	120.0 (3)	N5—C34—C33	121.3 (3)
N4—C6—C5	120.8 (3)	C35—C34—C33	116.4 (3)
N4—C6—C7	118.7 (3)	C36—C35—C34	118.8 (3)
C5—C6—C7	120.0 (3)	C36—C35—H35	120.6
C6—C7—C11	120.0 (3)	C34—C35—H35	120.6
C6—C7—C8	123.0 (3)	C35—C36—C37	119.1 (3)
C11—C7—C8	117.0 (3)	C35—C36—H36	120.5
C9—C8—C7	119.4 (3)	C37—C36—H36	120.5
C9—C8—H8	120.3	C45—C37—C38	120.2 (3)
C7—C8—H8	120.3	C45—C37—C36	117.5 (3)
C10—C9—C8	119.1 (3)	C38—C37—C36	122.3 (3)
C10—C9—H9	120.5	C39—C38—C37	121.6 (3)
C8—C9—H9	120.5	C39—C38—H38	119.2
N2—C10—C9	124.6 (3)	C37—C38—H38	119.2
N2—C10—H10	117.7	C38—C39—C40	118.8 (3)
C9—C10—H10	117.7	C38—C39—H39	120.6
C12—C11—C7	120.0 (3)	C40—C39—H39	120.6
C12—C11—N2	116.0 (3)	C39—C40—C46	120.6 (3)
C7—C11—N2	123.9 (3)	C39—C40—C41	123.7 (3)
N1—C12—C11	116.6 (3)	C46—C40—C41	115.8 (3)
N1—C12—C4	123.3 (3)	C42—C41—C40	120.4 (3)
C11—C12—C4	120.0 (3)	C42—C41—H41	119.8
N3—C13—C18	120.8 (3)	C40—C41—H41	119.8
N3—C13—C14	116.8 (3)	C41—C42—C43	120.5 (3)
C18—C13—C14	122.3 (3)	C41—C42—H42	119.7
C13—C14—C15	110.5 (3)	C43—C42—H42	119.7
C13—C14—H14A	109.5	N6—C43—C42	120.8 (3)
C15—C14—H14A	109.5	N6—C43—C44	120.6 (3)
C13—C14—H14B	109.5	C42—C43—C44	118.5 (3)
C15—C14—H14B	109.5	C43—C44—H44A	109.5
H14A—C14—H14B	108.1	C43—C44—H44B	109.5
C16—C15—C14	110.6 (3)	H44A—C44—H44B	109.5
C16—C15—H15A	109.5	C43—C44—H44C	109.5
C14—C15—H15A	109.5	H44A—C44—H44C	109.5
C16—C15—H15B	109.5	H44B—C44—H44C	109.5
C14—C15—H15B	109.5	N5—C45—C37	123.5 (3)
H15A—C15—H15B	108.1	N5—C45—C46	117.7 (3)
C15—C16—C17	113.8 (3)	C37—C45—C46	118.7 (3)
C15—C16—H16A	108.8	N6—C46—C45	116.5 (3)
C17—C16—H16A	108.8	N6—C46—C40	123.9 (3)
C15—C16—H16B	108.8	C45—C46—C40	119.5 (3)
C17—C16—H16B	108.8	O12—Cl1—O14	113.04 (19)
H16A—C16—H16B	107.7	O12—Cl1—O11	117.0 (2)
C18—C17—C16	112.8 (3)	O14—Cl1—O11	107.34 (18)
C18—C17—H17A	109.0	O12—Cl1—O13	108.69 (18)
C16—C17—H17A	109.0	O14—Cl1—O13	106.09 (19)
C18—C17—H17B	109.0	O11—Cl1—O13	103.79 (19)
C16—C17—H17B	109.0	O22—Cl2—O24	103.87 (19)
H17A—C17—H17B	107.8	O22—Cl2—O23	110.77 (19)
N4—C18—C13	123.2 (3)	O24—Cl2—O23	103.07 (19)
N4—C18—C17	114.7 (3)	O22—Cl2—O21	116.78 (19)
C13—C18—C17	122.0 (3)	O24—Cl2—O21	108.8 (2)
C20—C19—H19A	109.5	O23—Cl2—O21	112.3 (2)
C20—C19—H19B	109.5	C1—N1—C12	116.7 (3)
H19A—C19—H19B	109.5	C1—N1—Ru1	129.0 (2)
C20—C19—H19C	109.5	C12—N1—Ru1	114.2 (2)
H19A—C19—H19C	109.5	C10—N2—C11	115.7 (3)
H19B—C19—H19C	109.5	C10—N2—Ru1	130.2 (2)
N7—C20—C21	119.8 (3)	C11—N2—Ru1	113.85 (19)
N7—C20—C19	119.9 (3)	C13—N3—C5	117.2 (3)
C21—C20—C19	120.3 (3)	C18—N4—C6	116.6 (3)
C22—C21—C20	121.8 (3)	C34—N5—C45	118.3 (3)
C22—C21—H21	119.1	C34—N5—Ru1	131.0 (2)
C20—C21—H21	119.1	C45—N5—Ru1	110.4 (2)
C21—C22—C23	120.4 (3)	C43—N6—C46	118.0 (3)
C21—C22—H22	119.8	C43—N6—Ru1	131.2 (2)
C23—C22—H22	119.8	C46—N6—Ru1	109.8 (2)
C24—C23—C32	119.8 (3)	C20—N7—C32	118.8 (3)
C24—C23—C22	124.3 (3)	C20—N7—Ru1	131.0 (2)
C32—C23—C22	115.9 (3)	C32—N7—Ru1	109.8 (2)
C25—C24—C23	121.1 (3)	C29—N8—C31	117.6 (3)
C25—C24—H24	119.4	C29—N8—Ru1	131.2 (2)
C23—C24—H24	119.4	C31—N8—Ru1	110.8 (2)
C24—C25—C26	119.5 (3)	H1WD—O1W—H1WC	103.0
C24—C25—H25	120.3	H2WA—O2W—H2WB	118.8
C26—C25—H25	120.3	H3WA—O3W—H3WB	109.5
C31—C26—C27	117.7 (3)	H4WD—O4W—H4WC	109.5
C31—C26—C25	120.4 (3)	H5WD—O5W—H5WA	109.5
C27—C26—C25	121.8 (3)	N2—Ru1—N1	78.80 (10)
C28—C27—C26	118.7 (3)	N2—Ru1—N5	93.20 (10)
C28—C27—H27	120.6	N1—Ru1—N5	170.61 (10)
C26—C27—H27	120.6	N2—Ru1—N7	170.56 (10)
C27—C28—C29	120.1 (3)	N1—Ru1—N7	91.88 (10)
C27—C28—H28	120.0	N5—Ru1—N7	96.22 (10)
C29—C28—H28	120.0	N2—Ru1—N6	79.60 (10)
N8—C29—C28	121.6 (3)	N1—Ru1—N6	94.86 (10)
N8—C29—C30	121.7 (3)	N5—Ru1—N6	78.72 (10)
C28—C29—C30	116.6 (3)	N7—Ru1—N6	102.96 (10)
C29—C30—H30B	109.5	N2—Ru1—N8	97.99 (10)
C29—C30—H30C	109.5	N1—Ru1—N8	85.57 (10)
H30B—C30—H30C	109.5	N5—Ru1—N8	100.53 (10)
C29—C30—H30A	109.5	N7—Ru1—N8	79.58 (10)
H30B—C30—H30A	109.5	N6—Ru1—N8	177.40 (10)
			
N1—C1—C2—C3	1.0 (5)	C11—C12—N1—C1	−178.7 (3)
C1—C2—C3—C4	0.4 (4)	C4—C12—N1—C1	5.3 (5)
C2—C3—C4—C5	−178.9 (3)	C11—C12—N1—Ru1	5.5 (4)
C2—C3—C4—C12	1.0 (4)	C4—C12—N1—Ru1	−170.5 (2)
C12—C4—C5—N3	−173.1 (3)	C9—C10—N2—C11	0.8 (5)
C3—C4—C5—N3	6.8 (5)	C9—C10—N2—Ru1	−173.5 (2)
C12—C4—C5—C6	0.0 (5)	C12—C11—N2—C10	178.9 (3)
C3—C4—C5—C6	179.9 (3)	C7—C11—N2—C10	−4.9 (4)
N3—C5—C6—N4	0.8 (4)	C12—C11—N2—Ru1	−5.8 (4)
C4—C5—C6—N4	−172.2 (3)	C7—C11—N2—Ru1	170.4 (2)
N3—C5—C6—C7	173.0 (3)	C18—C13—N3—C5	5.8 (5)
C4—C5—C6—C7	0.0 (4)	C14—C13—N3—C5	−174.3 (3)
N4—C6—C7—C11	172.4 (3)	C4—C5—N3—C13	168.3 (3)
C5—C6—C7—C11	0.0 (4)	C6—C5—N3—C13	−4.8 (4)
N4—C6—C7—C8	−11.0 (4)	C13—C18—N4—C6	−1.3 (4)
C5—C6—C7—C8	176.7 (3)	C17—C18—N4—C6	176.0 (3)
C6—C7—C8—C9	178.0 (3)	C5—C6—N4—C18	2.2 (4)
C11—C7—C8—C9	−5.3 (4)	C7—C6—N4—C18	−170.0 (3)
C7—C8—C9—C10	1.7 (5)	C35—C34—N5—C45	−7.4 (5)
C8—C9—C10—N2	0.6 (5)	C33—C34—N5—C45	177.1 (3)
C6—C7—C11—C12	0.0 (4)	C35—C34—N5—Ru1	166.0 (3)
C8—C7—C11—C12	−176.9 (3)	C33—C34—N5—Ru1	−9.5 (5)
C6—C7—C11—N2	−176.1 (3)	C37—C45—N5—C34	7.9 (5)
C8—C7—C11—N2	7.1 (5)	C46—C45—N5—C34	−168.1 (3)
C7—C11—C12—N1	−176.2 (3)	C37—C45—N5—Ru1	−166.8 (3)
N2—C11—C12—N1	0.2 (4)	C46—C45—N5—Ru1	17.2 (3)
C7—C11—C12—C4	0.0 (5)	C42—C43—N6—C46	8.1 (4)
N2—C11—C12—C4	176.4 (3)	C44—C43—N6—C46	−170.3 (3)
C5—C4—C12—N1	175.9 (3)	C42—C43—N6—Ru1	−158.9 (2)
C3—C4—C12—N1	−4.0 (5)	C44—C43—N6—Ru1	22.7 (4)
C5—C4—C12—C11	0.0 (5)	C45—C46—N6—C43	169.8 (3)
C3—C4—C12—C11	−179.9 (3)	C40—C46—N6—C43	−6.4 (5)
N3—C13—C14—C15	154.5 (3)	C45—C46—N6—Ru1	−20.6 (3)
C18—C13—C14—C15	−25.6 (5)	C40—C46—N6—Ru1	163.2 (3)
C13—C14—C15—C16	54.4 (4)	C21—C20—N7—C32	−7.2 (4)
C14—C15—C16—C17	−59.8 (4)	C19—C20—N7—C32	171.1 (3)
C15—C16—C17—C18	33.0 (4)	C21—C20—N7—Ru1	164.5 (2)
N3—C13—C18—N4	−2.9 (5)	C19—C20—N7—Ru1	−17.2 (4)
C14—C13—C18—N4	177.2 (3)	C23—C32—N7—C20	8.9 (4)
N3—C13—C18—C17	−179.9 (3)	C31—C32—N7—C20	−170.9 (3)
C14—C13—C18—C17	0.1 (5)	C23—C32—N7—Ru1	−164.4 (2)
C16—C17—C18—N4	179.6 (3)	C31—C32—N7—Ru1	15.7 (3)
C16—C17—C18—C13	−3.2 (4)	C28—C29—N8—C31	4.9 (5)
N7—C20—C21—C22	0.3 (5)	C30—C29—N8—C31	−175.7 (3)
C19—C20—C21—C22	−178.0 (3)	C28—C29—N8—Ru1	−166.8 (3)
C20—C21—C22—C23	5.1 (5)	C30—C29—N8—Ru1	12.5 (5)
C21—C22—C23—C24	175.6 (3)	C26—C31—N8—C29	−7.1 (4)
C21—C22—C23—C32	−3.4 (5)	C32—C31—N8—C29	168.8 (3)
C32—C23—C24—C25	5.2 (5)	C26—C31—N8—Ru1	166.3 (2)
C22—C23—C24—C25	−173.8 (3)	C32—C31—N8—Ru1	−17.8 (3)
C23—C24—C25—C26	−2.4 (5)	C10—N2—Ru1—N1	−179.0 (3)
C24—C25—C26—C31	−3.0 (5)	C11—N2—Ru1—N1	6.6 (2)
C24—C25—C26—C27	173.6 (3)	C10—N2—Ru1—N5	5.9 (3)
C31—C26—C27—C28	0.5 (4)	C11—N2—Ru1—N5	−168.4 (2)
C25—C26—C27—C28	−176.2 (3)	C10—N2—Ru1—N6	83.8 (3)
C26—C27—C28—C29	−2.5 (5)	C11—N2—Ru1—N6	−90.5 (2)
C27—C28—C29—N8	−0.3 (5)	C10—N2—Ru1—N8	−95.2 (3)
C27—C28—C29—C30	−179.7 (3)	C11—N2—Ru1—N8	90.5 (2)
C27—C26—C31—N8	4.4 (4)	C1—N1—Ru1—N2	178.3 (3)
C25—C26—C31—N8	−178.9 (3)	C12—N1—Ru1—N2	−6.6 (2)
C27—C26—C31—C32	−171.5 (3)	C1—N1—Ru1—N7	−0.2 (3)
C25—C26—C31—C32	5.2 (4)	C12—N1—Ru1—N7	175.0 (2)
C24—C23—C32—N7	177.3 (3)	C1—N1—Ru1—N6	−103.3 (3)
C22—C23—C32—N7	−3.6 (4)	C12—N1—Ru1—N6	71.8 (2)
C24—C23—C32—C31	−2.8 (4)	C1—N1—Ru1—N8	79.2 (3)
C22—C23—C32—C31	176.3 (3)	C12—N1—Ru1—N8	−105.6 (2)
N8—C31—C32—N7	1.3 (4)	C34—N5—Ru1—N2	−116.3 (3)
C26—C31—C32—N7	177.5 (3)	C45—N5—Ru1—N2	57.4 (2)
N8—C31—C32—C23	−178.5 (3)	C34—N5—Ru1—N7	62.9 (3)
C26—C31—C32—C23	−2.4 (4)	C45—N5—Ru1—N7	−123.3 (2)
N5—C34—C35—C36	1.0 (6)	C34—N5—Ru1—N6	164.9 (3)
C33—C34—C35—C36	176.7 (4)	C45—N5—Ru1—N6	−21.3 (2)
C34—C35—C36—C37	5.0 (6)	C34—N5—Ru1—N8	−17.6 (3)
C35—C36—C37—C45	−4.5 (6)	C45—N5—Ru1—N8	156.2 (2)
C35—C36—C37—C38	176.8 (4)	C20—N7—Ru1—N1	−106.1 (3)
C45—C37—C38—C39	4.6 (5)	C32—N7—Ru1—N1	66.2 (2)
C36—C37—C38—C39	−176.8 (4)	C20—N7—Ru1—N5	69.2 (3)
C37—C38—C39—C40	2.8 (5)	C32—N7—Ru1—N5	−118.6 (2)
C38—C39—C40—C46	−7.5 (5)	C20—N7—Ru1—N6	−10.6 (3)
C38—C39—C40—C41	173.5 (3)	C32—N7—Ru1—N6	161.61 (19)
C39—C40—C41—C42	−176.0 (3)	C20—N7—Ru1—N8	168.8 (3)
C46—C40—C41—C42	4.9 (5)	C32—N7—Ru1—N8	−18.96 (19)
C40—C41—C42—C43	−3.4 (5)	C43—N6—Ru1—N2	94.9 (3)
C41—C42—C43—N6	−3.4 (5)	C46—N6—Ru1—N2	−72.9 (2)
C41—C42—C43—C44	175.0 (3)	C43—N6—Ru1—N1	17.2 (3)
C38—C37—C45—N5	176.7 (3)	C46—N6—Ru1—N1	−150.6 (2)
C36—C37—C45—N5	−2.0 (5)	C43—N6—Ru1—N5	−169.7 (3)
C38—C37—C45—C46	−7.3 (5)	C46—N6—Ru1—N5	22.49 (19)
C36—C37—C45—C46	174.0 (3)	C43—N6—Ru1—N7	−75.9 (3)
N5—C45—C46—N6	2.5 (4)	C46—N6—Ru1—N7	116.4 (2)
C37—C45—C46—N6	−173.8 (3)	C29—N8—Ru1—N2	21.4 (3)
N5—C45—C46—C40	178.9 (3)	C31—N8—Ru1—N2	−150.76 (19)
C37—C45—C46—C40	2.7 (5)	C29—N8—Ru1—N1	99.5 (3)
C39—C40—C46—N6	−179.2 (3)	C31—N8—Ru1—N1	−72.7 (2)
C41—C40—C46—N6	−0.1 (5)	C29—N8—Ru1—N5	−73.3 (3)
C39—C40—C46—C45	4.7 (5)	C31—N8—Ru1—N5	114.5 (2)
C41—C40—C46—C45	−176.2 (3)	C29—N8—Ru1—N7	−167.8 (3)
C2—C1—N1—C12	−3.7 (5)	C31—N8—Ru1—N7	20.00 (19)
C2—C1—N1—Ru1	171.3 (2)		
